# Role of ferroptosis in hypoxic preconditioning to reduce propofol neurotoxicity

**DOI:** 10.3389/fphar.2023.1121280

**Published:** 2023-02-02

**Authors:** Jing Chen, Fei Xiao, Lifei Chen, Zhan Zhou, Yi Wei, Yu Zhong, Li Li, Yubo Xie

**Affiliations:** ^1^ Department of Anesthesiology, The First Affiliated Hospital of Guangxi Medical University, Nanning, China; ^2^ Guangxi Key Laboratory of Enhanced Recovery After Surgery for Gastrointestinal Cancer, The First Affiliated Hospital of Guangxi Medical University, Nanning, China

**Keywords:** propofol, neurotoxicity, ferroptosis, hypoxic preconditioning, hippocampus

## Abstract

**Background:** An increasing number of studies have reported that neurotoxicity of propofol may cause long-term learning and cognitive dysfunction. Hypoxic preconditioning has been shown to have neuroprotective effects, reducing the neurotoxicity of propofol. Ferroptosis is a new form of death that is different from apoptosis, necrosis, autophagy and pyroptosis. However, it is unclear whether hypoxic preconditioning reduces propofol neurotoxicity associated with ferroptosis. Thus, we aimed to evaluate the effect of propofol on primary hippocampal neurons *in vitro* to investigate the neuroprotective mechanism of hypoxic preconditioning and the role of ferroptosis in the reduction of propofol neurotoxicity by hypoxic preconditioning.

**Methods:** Primary hippocampal neurons were cultured for 8 days *in vitro* and pretreated with or without propofol, hypoxic preconditioning, agonists or inhibitors of ferroptosis. Cell counting kit-8, Calcein AM, Reactive oxygen species (ROS), Superoxide dismutase (SOD), Ferrous iron (Fe^2+^), Malondialdehyde (MDA) and Mitochondrial membrane potential assay kit with JC-1 (JC-1) assays were used to measure cell viability, Reactive oxygen species level, Superoxide dismutase content, Fe^2+^ level, MDA content, and mitochondrial membrane potential. Cell apoptosis was evaluated using flow cytometry analyses, and ferroptosis-related proteins were determined by Western blot analysis.

**Results:** Propofol had neurotoxic effects that led to decreased hippocampal neuronal viability, reduced mitochondrial membrane potential, decreased SOD content, increased ROS level, increased Fe^2+^ level, increased MDA content, increased neuronal apoptosis, altered expression of ferroptosis-related proteins and activation of ferroptosis. However, hypoxic preconditioning reversed these effects, inhibited ferroptosis caused by propofol and reduced the neurotoxicity of propofol.

**Conclusion:** The neurotoxicity of propofol in developing rats may be related to ferroptosis. Propofol may induce neurotoxicity by activating ferroptosis, while hypoxic preconditioning may reduce the neurotoxicity of propofol by inhibiting ferroptosis.

## Introduction

Propofol is the most commonly used short-acting intravenous anesthetic in the clinic and is widely used in infant anesthesia. Our previous studies have shown that propofol has neurotoxic effects in the developing brain, and this toxic effect may lead to long-term learning and memory dysfunction (Zhong et al., 2014; Zhong et al., 2018). At the same time, we also found that dexmedetomidine, a highly selective alpha-2 adrenoceptor agonist, reduces the neurotoxicity of propofol and has a neuroprotective effect (Xiao et al., 2018; Tu et al., 2019). However, it is not advisable to use one drug to mitigate the toxic effects of another. Therefore, we investigated a method to reverse the neurotoxicity of propofol that was simple and easy to implement in the clinic but was not a drug.

Hypoxic preconditioning is a biological method that increases the resistance or tolerance of tissues or organisms to subsequent damage resulting from temporary and moderate hypoxia in advance. Thus, hypoxic preconditioning produces the preadaptation of hypoxia in tissues to protect the tissues from subsequent damage. Hypoxic preconditioning has been demonstrated to play a protective role not only in the cardiovascular and urinary systems (Zhang C. S. et al., 2019; Torosyan et al., 2021) but also in the nervous system (Correia et al., 2022; Pang et al., 2022). Our previous studies have shown that hypoxic preconditioning reduces the neurotoxicity of propofol, reduces the rate of neuronal apoptosis and promotes the expression of protective proteins in the nervous system, thereby maintaining the integrity of neuronal function (Lv et al., 2018; Xiao et al., 2020). Therefore, the neuroprotective effect of hypoxic preconditioning in cerebral ischemia and cerebral trauma has been confirmed (Shu et al., 2016; Zhan et al., 2017; Chao et al., 2020; Liu et al., 2021). Although the mechanism of hypoxic preconditioning is not well clear, the research focus is mainly on endogenous signal cascade reactions.

Iron is one of the most abundant and indispensable trace elements in the body, and it has many important physiological functions. Iron is the main raw material of hemoglobin and myoglobin, and it not only participates in the biosynthesis of DNA and ATP but is also an important electron transport chain and metalloproteinase cofactor in mitochondria (Tang et al., 2021). Therefore, iron balance is essential for maintaining the health of the body. Ferroptosis, as discovered by Dolma (Dolma et al., 2003) and named by Dixon (Dixon et al., 2012), is a new type of iron-dependent programmed cell death that is different from apoptosis, necrosis, autophagy and pyroptosis. The essence of ferroptosis is the disturbance of intracellular lipid oxide metabolism, which occurs under the catalytic effect of iron ions. When the antioxidant capacity of cells is weakened, Reactive oxygen species (ROS) accumulate, resulting in an imbalance in the intracellular redox reaction and ultimately inducing cell death (Xu et al., 2020).

It is unclear whether the neurotoxicity of propofol is associated with ferroptosis. In the present study, primary hippocampal neurons of rats were used to investigate the effect of propofol on primary hippocampal neurons *in vitro*, the neuroprotective mechanism of hypoxia preconditioning and the role of ferroptosis in reducing the neurotoxicity of propofol by hypoxia preconditioning.

## Materials and methods

### Materials

Trypsin Digestion solutions (0.25%, without phenol red) (Beijing Solarbio Science and Technology Co., Ltd., Beijing, China, Cat#T1350), Poly-L-lysine (Sigma-Aldrich Chemical Co., St Louis, MO, United States, Cat#P4832), Penicillin-Streptomycin Liquid (100×, Beijing Solarbio Science & Technology Co., Beijing, China, Cat#P1400), L-Glutamine (Sigma-Aldrich Chemical Co., St Louis, MO, United States, Cat#G7513), Gibco^TM^ FBS (Thermo Fisher Scientific Inc., Auckland, New Zealand, Cat#10091148), Gibco^TM^ BebchStable DMEM/F12 (Thermo Fisher Scientific Inc., Grand Island, NY, United States, Cat#A4192001), Gibco^TM^ B-27 (50×, Thermo Fisher Scientific Inc., Grand Island, NY, Cat#17504044), Paraformaldehyde (4%, Beijing Solarbio Science & Technology Co., Beijing, China, Cat#P1110), PBS (Beijing Solarbio Science & Technology Co., Beijing, China, Cat#P1020), Triton X-100 (Beijing Solarbio Science & Technology Co., Beijing, China, Cat#P1080), Hypoxia Incubator Chamber (NovoBiotechnology Co.,. Ltd., Beijing, China, NO. 504001), Annexin V Apoptosis Detection Kit I (BD Biosciences, New Jersey, NY, Cat# 556547), CCK-8 Cell Proliferation and Cytotoxicity Assay Kit (Beijing Solarbio Science & Technology Co., Beijing, China, Cat#CA1210), Calcein AM Cell Viability Assay Kit (Beyotime Biotechnology, Shanghai, China, Cat#C2013S), Mitochondrial membrane potential assay kit with JC-1 (Beyotime Biotechnology, Shanghai, China, Cat#C2006), ATP Assay Kit (Beyotime Biotechnology, Shanghai, China, Cat#S2006), Total Superoxide Dismutase (T-SOD) assay Kit (Nanjing Jiancheng Bioengineering Institute, Nanjing, China, Cat#A001-1-1), Malondialdehyde (MDA) Assay Kit (Beijing Solarbio Science & Technology Co., Beijing, China, Cat#BC0025), ROS Assay Kit (Beyotime Biotechnology, Shanghai, China, Cat#S0033S), Iron Assay Kit (Sigma-Aldrich, United States, Cat#MAK025), Polyvinylidene difluoride (PVDF) membrane (0.22 µm, EMD Millipore, Billerica, MA, United States), Bovine serum albumin (BSA) (Beijing Solarbio Science & Technology Co., Beijing, China, Cat#SW3015), TBST (Beijing Solarbio Science & Technology Co., Ltd., Beijing, China, Cat#T1085), RIPA Lysis Buffer (Beyotime Biotechnology, Shanghai, China, Cat#P0013B), Phenylmethanesulfonyl fluoride (PMSF) Solution (Beyotime Biotechnology, Shanghai, China, Cat#ST507), Protease and phosphatase inhibitor cocktail (Beyotime Biotechnology, Shanghai, China, Cat#P1045). Microtubule-associated protein 2 (MAP2) (Abcam, Cambridge, England, UK, Cat#ab183830), Transferrin receptor protein 1 (TFR1) (Beijing Bioss Biotechnology Co., Ltd., Beijing, China, Cat#bsm-54633R), Divalent metal transporter 1 (DMT1) (Proteintech, Chicago, IL, United States, Cat#20507-1-AP), Glutathione peroxidase 4 (GPX4) (Cell Signaling Technology, United States, Cat#59735), Solute carrier family seven member 11 (SLC7A11) (Abcam, United States, Cat#ab175186), Ferroportin 1 (FPN1) (Proteintech, Chicago, IL, United States, Cat#26601-1-AP), β-actin (Abcam, United States, Cat#ab8227), LI-COR fluorescent secondary antibody (LI-COR, Lincoln, NE, United States).

### Hippocampal neuron culture

All experimental procedures in this study were approved by The Animal Care & Welfare Committee of Guangxi Medical University (No. scxk GUI 2014-0002) and implemented in accordance with the guidelines for the ethical review of laboratory animal welfare (GB/T 35892-2018). The 16- to 18-day-old Sprague-Dawley (SD) pregnant rats from the experimental animal center of Guangxi Medical University. Primary hippocampal neurons were isolated from 16- to 18-day-old SD pregnant rat fetuses according to previous experimental methods (Tu et al., 2019; Wei et al., 2021). Under sterile conditions, the uterus was exposed rapidly to remove the fetus after anesthetization of pregnant rats with 2% sevoflurane. The fetus was decapitated after anesthetization, and the hippocampus was exposed and separated. The hippocampal tissue was shredded by ophthalmic forceps into a tissue fragment suspension with a size of l mm × l mm × l mm. The hippocampal tissue fragment suspension was treated with an equal volume of 0.25% trypsin digestion solution and digested at 37°C for 15 min. An equal volume of plating medium was then added to the hippocampal tissue fragment suspension to terminate the digestion. After digestion, the upper layer was discarded after centrifugation at 4,000 g/min for 5 min at 37°C. An appropriate amount of cell culture medium was added to the tissue, and the tissue was dissociated by a glass pipette to obtain a single-cell suspension. The single-cell suspension was seeded onto poly-L-lysine-coated plates at a density of 1-2×10^6^ cells/ml in plating medium containing 1% penicillin-streptomycin, 1% L-glutamine, 10% FBS and 88% Gibco BebchStable^TM^ DMEM/F12 at 37°C in a humidified atmosphere of 5% CO_2_ and 95% air. After 4 h, the medium was replaced with serum-free maintenance medium. The maintenance medium consisted of 96% neurobasal medium, 2% B-27, 1% 200 mM glutamine and 1% penicillin/streptomycin. The medium was replaced every 2 days, and half of the medium was replaced each time. Cell growth, morphology, density and protrusion were observed daily, and all experiments were performed at 8 days *in vitro* (8 DIV).

MAP2 is a phosphoprotein that mainly exists in the cell bodies, dendrites and dendritic spines of neurons in normal brain tissue, and it is one of the most abundant proteins in the brain. After discarding the medium, hippocampal neurons (8 DIV) were washed three times with PBS (5 min each), fixed with 4% paraformaldehyde for 30 min at room temperature, washed three times (8 min each), permeabilized with 0.1% Triton X-100 for 1 h at room temperature and incubated with a primary antibody against MAP2 (dilution 1:250) overnight at 4°C. The neurons were then washed three times with PBS (5 min each), incubated with a horseradish peroxidase-labeled secondary antibody for 10 min at room temperature, stained with hematoxylin, sealed with neutral glue and observed using a laser microscope.

### Hypoxic preconditioning

The hippocampal neurons (8 DIV) were placed into a hypoxia incubator chamber. Part of the chamber was submerged in a 42°C water bath to maintain a constant thermal environment at 36°C ambient temperature, and the oxygen concentration was regulated in the hypoxia incubator chamber. The neurons were exposed to five cycles of hypoxia (1% O_2_/10% CO_2_/89% N_2_) for 20 min and normoxia (5% CO_2_ + 95% air) for 20 min, after which the neurons were placed in an atmosphere of 5% CO_2_ and 95% air for 2 h (Wu et al., 2005).

### Experimental groups and treatment

**FIGURE 1:**
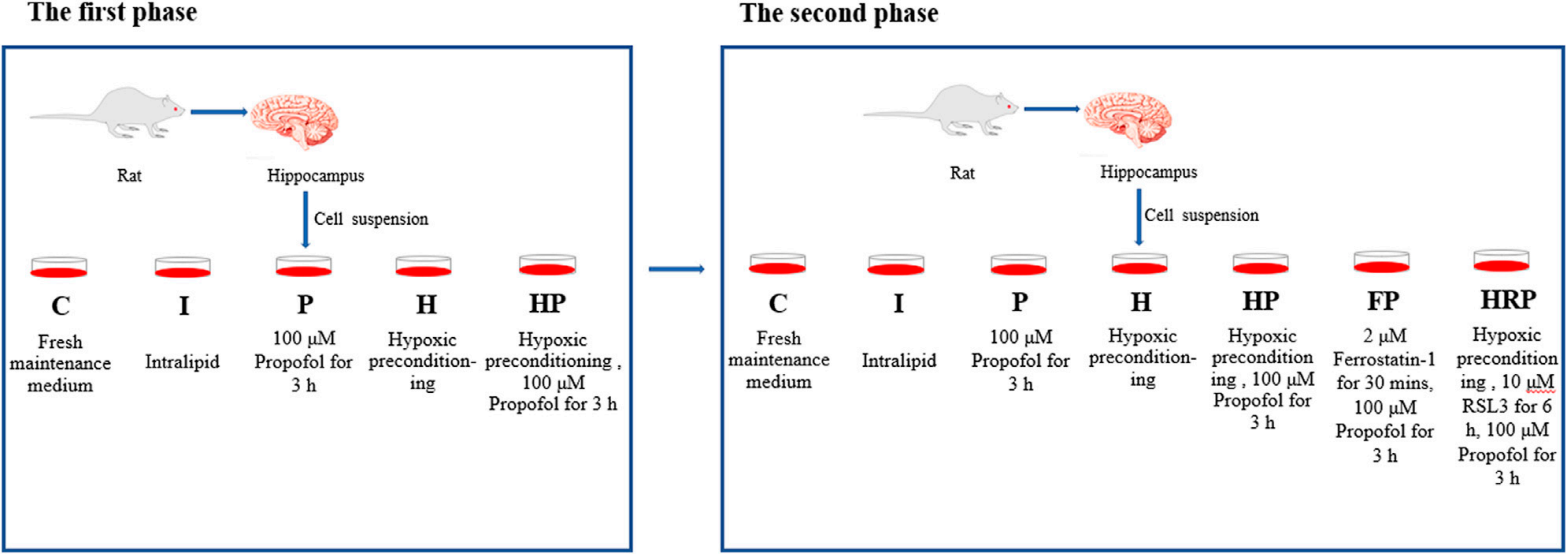
Experimental groups and treatments. In the first phase of the research, the cells in Group C and Group I were incubated with fresh maintenance medium and intralipid vehicle, respectively. The cells in Group P were incubated with 100 μM propofol for 3 h. Cells in Group H were treated with hypoxic preconditioning, and cells in Group HP were subjected to hypoxic preconditioning before incubation with propofol. In the second phase of the research, the cells in Group C, Group I, Group P, Group H and Group HP were processed as the first phase of the study. The cells in Group FP were treated with 2 μM ferrostatin-1 for 30 min before incubation with propofol. The cells in the HRP group were treated with 10 μM RSL3 for 6 h after hypoxic preconditioning and then incubated with 100 μM propofol for 3 h.

In the first phase of the study, primary hippocampal neurons (8 DIV) were randomly divided into the following five groups using a randomization table: Group C (control group), Group I (intralipid vehicle group), Group P (propofol group), Group H (hypoxic preconditioning group) and Group HP (hypoxic preconditioning + propofol group). The cells in Group C and Group I were incubated with fresh maintenance medium and intralipid vehicle, respectively. The cells in Group P were treated with 100 μM propofol for 3 h. Cells in Group H were treated with hypoxic preconditioning, and cells in Group HP were given hypoxic preconditioning before incubation with propofol (Figure 1).

In the second phase of the study, primary hippocampal neurons (8 DIV) were randomly divided into the following seven groups: Group C (control group), Group I (intralipid vehicle group), Group P (propofol group), Group H (hypoxic preconditioning group), Group HP (hypoxic preconditioning + propofol group), Group FP (ferrostatin-1 + propofol group) and Group HRP (hypoxic preconditioning + propofol + RSL3 group). The cells in Group C, Group I, Group P, Group H and Group HP were processed the same as the first phase of the study. The cells in Group FP were given 2 μM ferrostatin-1 for 30 min (Huang et al., 2022) before incubation with propofol. The cells in Group HRP were given 10 μM RSL3 for 6 h (Zhang Y et al., 2019) after hypoxic preconditioning followed by incubation with 100 μM propofol for 3 h (Figure 1).

### Evaluation of neuronal cell viability, mitochondrial membrane potential, ATP, SOD, MDA, ROS, and Fe^2+^

Hippocampal neuron viability was measured by a cell counting kit-8 (CCK-8) and Calcein-AM assay kit. The levels of ATP, ROS, MDA, SOD and Fe^2+^ were measured by ATP, ROS, MDA, SOD and Fe^2+^ kits, respectively, and mitochondrial membrane potential was measured by a JC-1 assay kits. Neuronal cell death was analyzed by flow cytometry, and the expression of ferroptosis-related proteins was determined by Western blotting. All kits were used according to the manufacturer’s instructions.

### Flow cytometry analysis

Neuronal cells from each group were harvested, digested with trypsin, and centrifuged at 1,000 rpm for 3 min at 4°C. The upper layer was discarded after centrifugation, and the pellet was resuspended in 200 μL of binding buffer. Next, 5 μL of Annexin V-FITC was added followed by incubation for 10 min, and 5 μL of propidium iodide was added followed by incubation for 5 min. Apoptosis was analyzed by a FACSCalibur flow cytometer (BD Biosciences).

### Western blot analysis

Neuronal cells from each group were harvested and lysed with cell lysis buffer (RIPA:PMSF:Protease and phosphatase inhibitor cocktail = 98:1:1) to extract total proteins. After determining the protein concentration, the proteins were electrophoresed on a 12% SDS‒PAGE gel and transferred to a PVDF membrane. The membrane was blocked with 5% BSA at 4°C for 1 h and incubated overnight at 4°C with the following antibodies: TFR1 (1:200), DMT1 (1:300), GPX4 (1:400), SLC7A11 (1:500), FPN1 (1:200) and β-actin (1:500). The membranes were then washed three times with TBST (5 min each) and incubated with the secondary antibody (1:10,000) at 4°C for 1 h. Finally, the membranes were scanned by an Odyssey system (LI-COR Biosciences).

### Statistical analysis

SPSS 24.0 (IBM, Armonk, NY, United States) and GraphPad 6.02 (OriginLab, Northampton, MA, United States) were used for all statistical analyses. The *t* test was used to analyze the data of two groups, and one-way ANOVA was used to analyze several groups. A *p-value* < 0.05 was considered a statistically significant difference.

## Results

### Hypoxic preconditioning alleviates propofol-induced hippocampal neuroapoptosis and promotes survival

**FIGURE 2:**
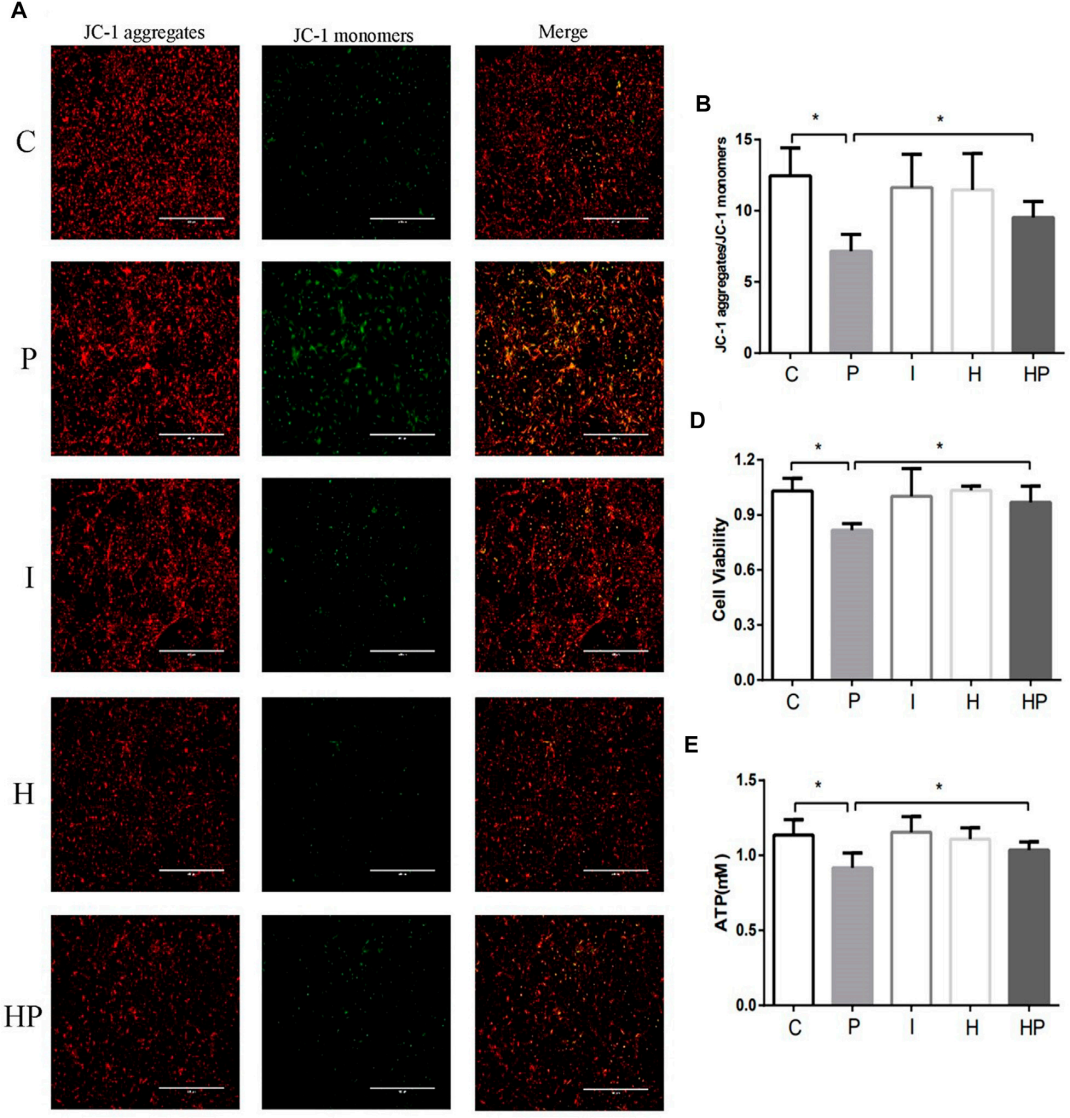
Changes in mitochondrial membrane potential, neuronal viability and ATP in primary hippocampal neurons exposed to propofol with or without hypoxic preconditioning. **(A,B)** Fluorescence images of JC-1 aggregates and JC-1 monomers indicating the mitochondrial membrane potential in each group (one-way ANOVA, *p < 0.05). **(D,E)** Cell viability and ATP content in each group (one-way ANOVA, *p < 0.05).

**FIGURE 3:**
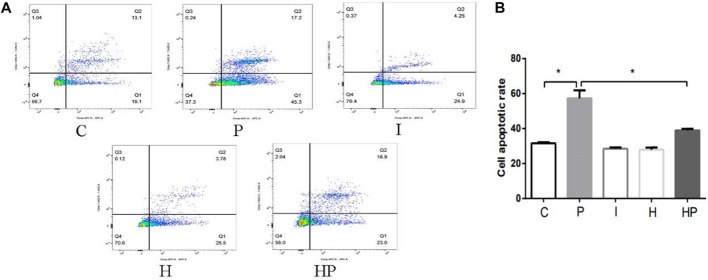
Changes in cell apoptosis in primary hippocampal neurons exposed to propofol with or without hypoxic preconditioning. **(A,B)** Cell apoptosis in each group (one-way ANOVA, *p < 0.05).

**FIGURE 4:**
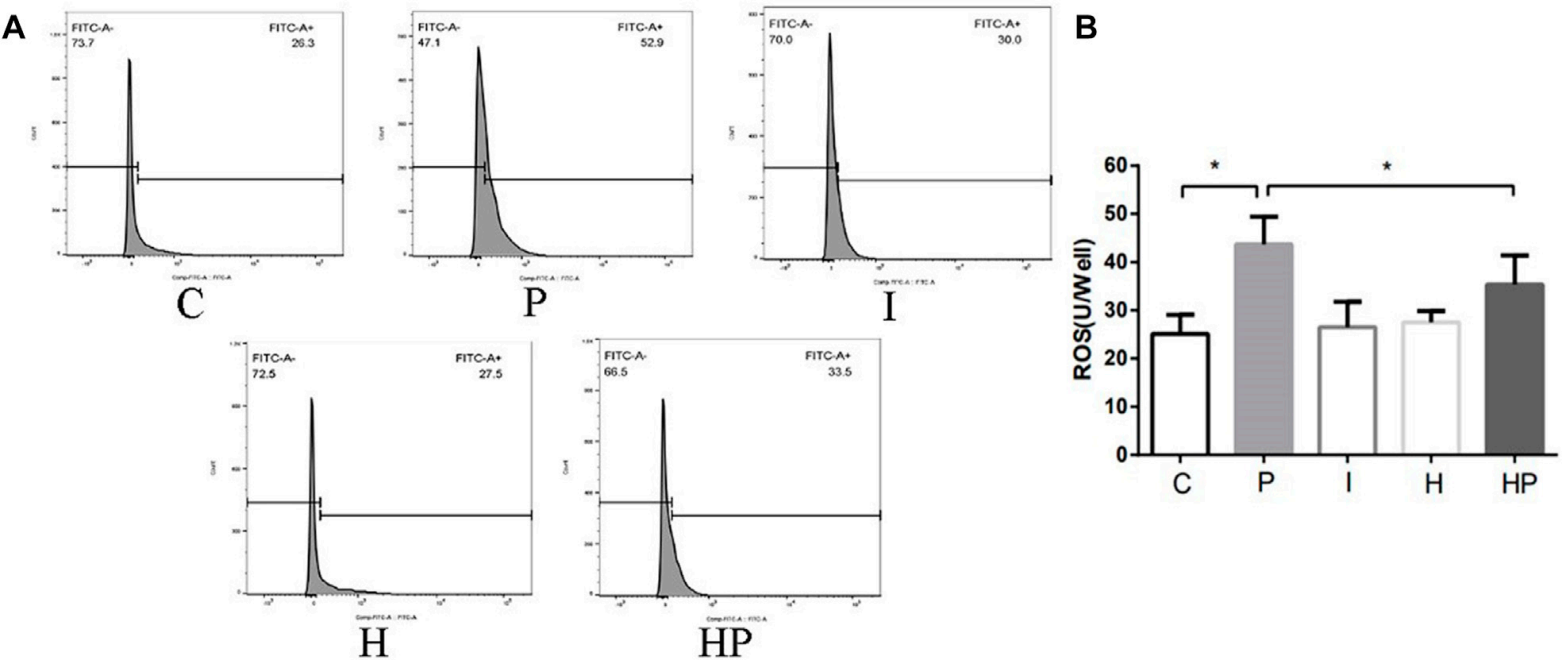
Changes in ROS in primary hippocampal neurons exposed to propofol with or without hypoxic preconditioning. **(A,B)** ROS content in each group (one-way ANOVA, *p < 0.05).

Compared to Group C, there were no significant differences in hippocampal neuronal viability, apoptosis, mitochondrial membrane potential, ATP content or ROS level in Group I and Group H (Figures 2–4). In contrast, treatment with 100 μM propofol for 3 h significantly reduced hippocampal neuronal viability, mitochondrial membrane potential and ATP content as well as increased ROS content and apoptosis in Group P (Figures 2–4). Furthermore, compared to Group P, hypoxic preconditioning before treatment with propofol significantly increased hippocampal neuronal viability, mitochondrial membrane potential and ATP content as well as significantly reduced the ROS content and apoptosis in Group HP (Figures 2–4). These findings demonstrated that propofol may damage hippocampal neurons and induce developmental neurotoxicity but that hypoxic preconditioning reduces this damage.

### Hypoxic preconditioning mitigates the effects of propofol on DMT1, TFR1, GPX4, SLC7A11 and FPN1 proteins in primary hippocampal neurons

**FIGURE 5:**
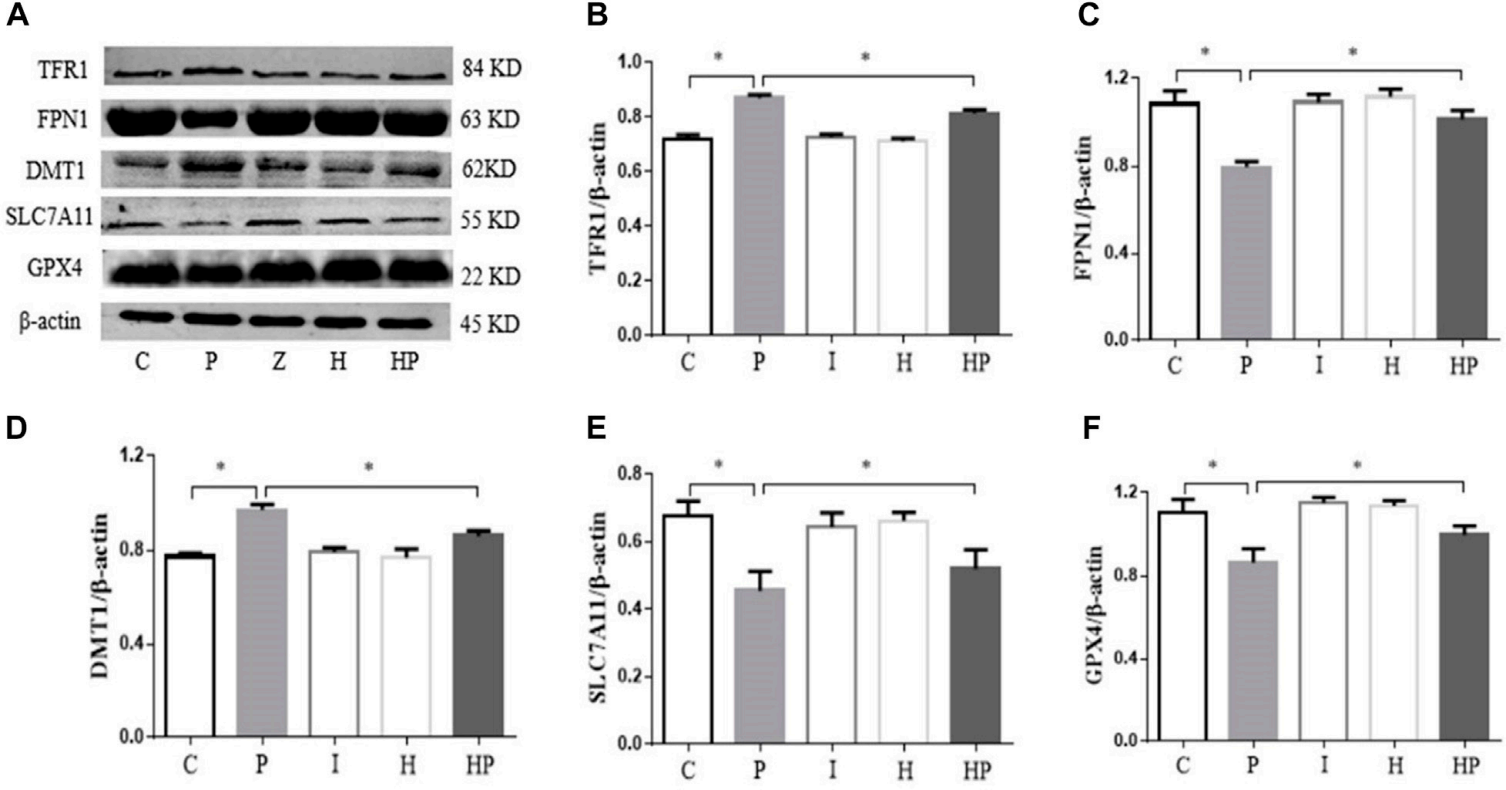
Protein expression levels of TFR1, FPN1, DMT1, SLC7A11 and GPX4 in primary hippocampal neurons exposed to propofol with or without hypoxic preconditioning. **(A)** TFR1, FPN1, DMT1, SLC7A11 and GPX4 protein expression was detected by Western blot analysis. **(B–F)** Quantitative analysis of the ratio of TFR1, FPN1, DMT1, SLC7A11 and GPX4 to β-actin (one-way ANOVA, *p < 0.05).

Western blot analysis demonstrated that 100 μM propofol significantly increased the protein expression of TFR1 and DMT1 but decreased the protein expression of GPX4, SLC7A11 and FPN1 in Group P compared to Group C (Figure 5). There were no significant differences in the protein expression levels of DMT1, TFR1, GPX4, SLC7A11 and FPN1 in Group I and Group H (Figure 5). However, after hypoxic preconditioning and propofol treatment, the protein expression of TFR1 and DMT1 was significantly decreased, whereas the protein expression of GPX4, SLC7A11 and FPN1 was significantly increased in Group HP compared to Group P (Figure 5).

### Propofol activates ferroptosis to produce neurotoxicity, while hypoxic preconditioning inhibits ferroptosis caused by propofol, thereby alleviating the neurotoxicity of propofol

**FIGURE 6:**
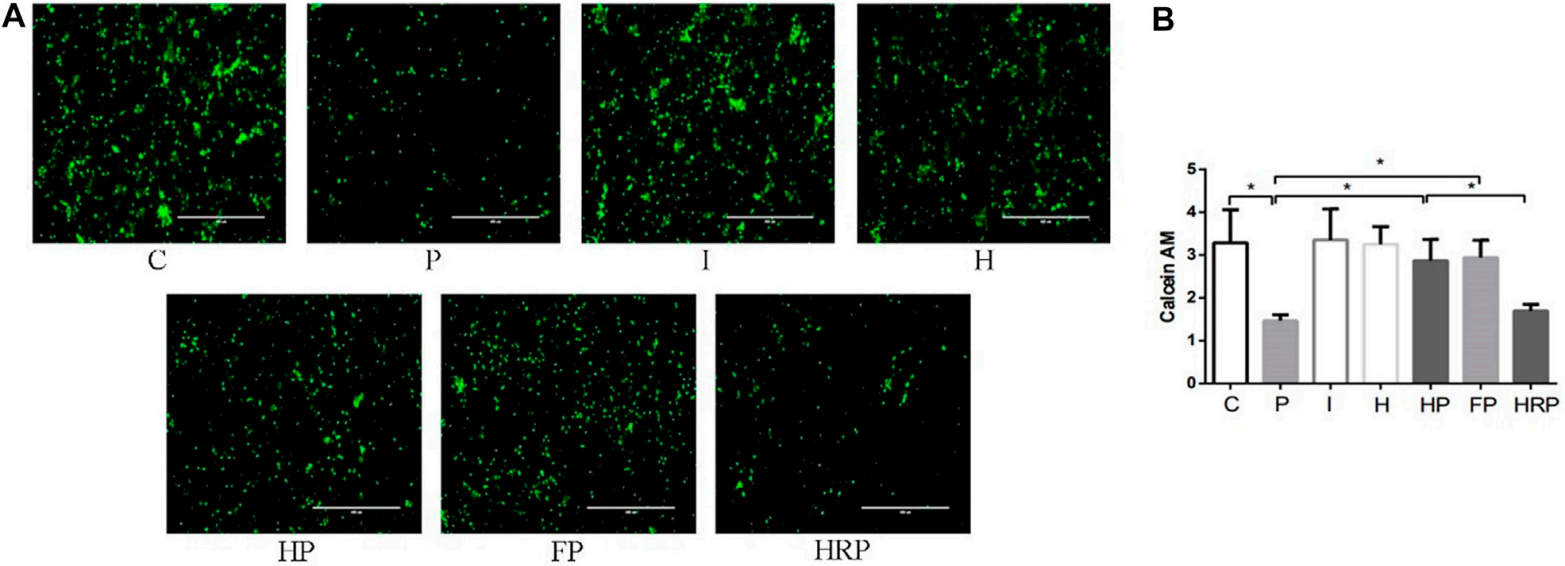
Cell viability in primary hippocampal neurons exposed to propofol with or without hypoxic preconditioning, inhibitor or agonist. **(A,B)** Calcein AM in each group (one-way ANOVA, *p < 0.05).

**FIGURE 7:**
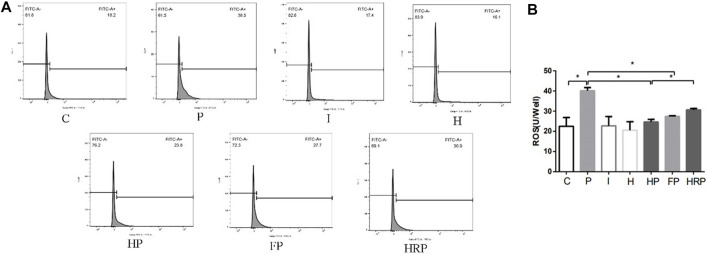
Changes in ROS in primary hippocampal neurons exposed to propofol with or without hypoxic preconditioning, inhibitor or agonist. **(A,B)** ROS content in each group (one-way ANOVA, *p < 0.05).

**FIGURE 8:**
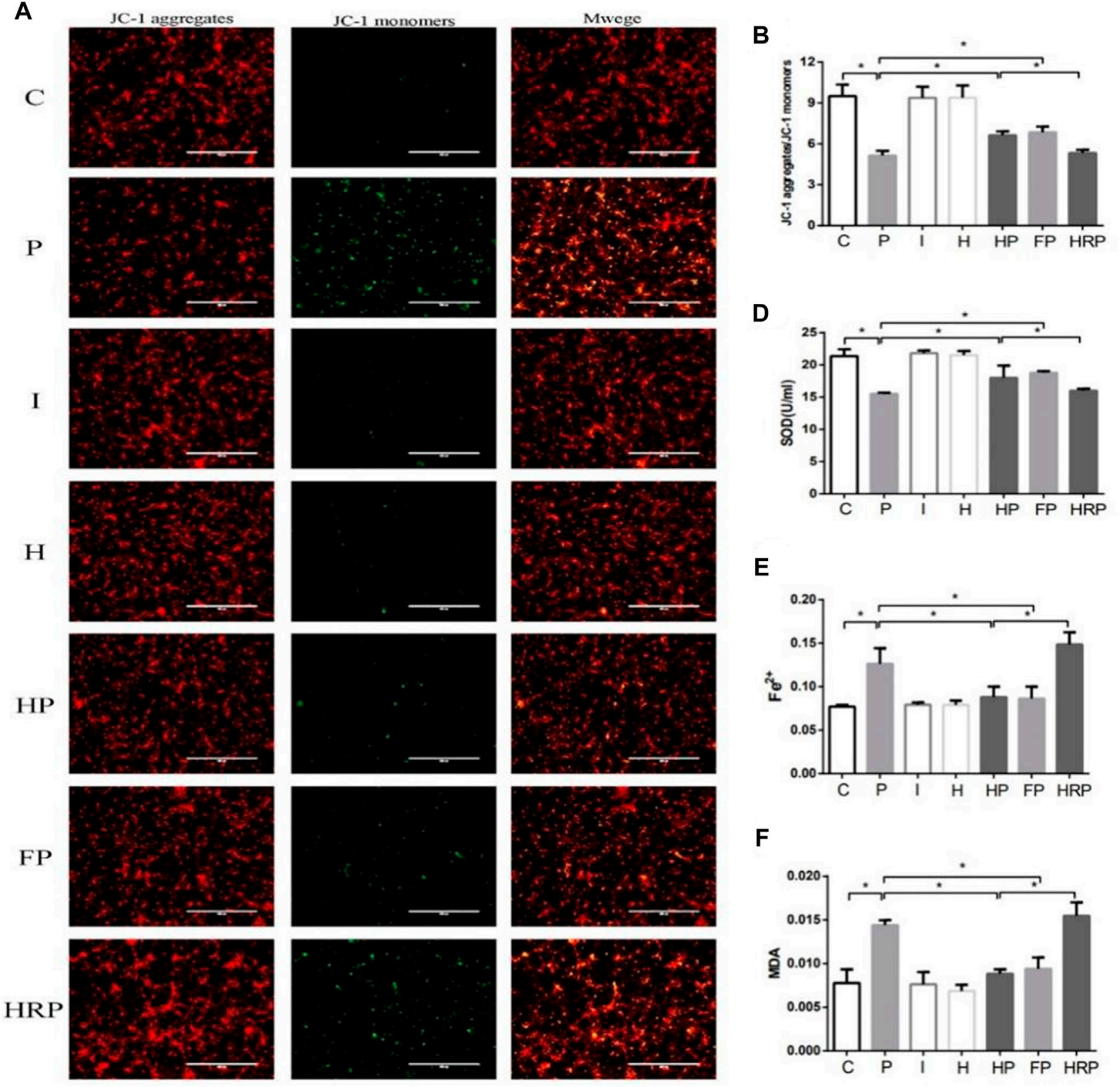
Changes in mitochondrial membrane potential, SOD level, Fe2+ level and MDA content in primary hippocampal neurons exposed to propofol with or without hypoxic preconditioning, inhibitor or agonist. **(A,B)** Fluorescence images of JC-1 aggregates and JC-1 monomers indicating the mitochondrial membrane potential in each group (one-way ANOVA, *p < 0.05). **(D–F)** The contents of SOD, Fe2+ and MDA in each group (one-way ANOVA, *p < 0.05).

There were no significant differences in hippocampal neuronal viability, mitochondrial membrane potential, SOD content, ROS level, Fe^2+^ level or MDA content in Group I or Group H compared to Group C (Figures 6–8). In contrast, treatment with 100 μM propofol for 3 h significantly reduced hippocampal neuronal viability, mitochondrial membrane potential and SOD content as well as increased the contents of ROS, Fe^2+^ and MDA in Group P (Figures 6–8). Compared to Group P, hypoxic preconditioning or pretreatment with ferroptosis inhibitors (ferrostatin-1, 2 μM) followed by propofol treatment significantly increased hippocampal neuronal viability, mitochondrial membrane potential and SOD content as well as significantly reduced the ROS, Fe^2+^ and MDA contents in Groups HP and FP (Figures 6–8). However, a ferroptosis agonist (RSL3, 10 μM) reversed the effects of hypoxic preconditioning. Compared to Group HP, the hippocampal neuronal viability, mitochondrial membrane potential and SOD content were significantly reduced in Group HRP, and the ROS, Fe^2+^ and MDA contents were significantly increased in Group HRP group (Figures 6–8).

**FIGURE 9:**
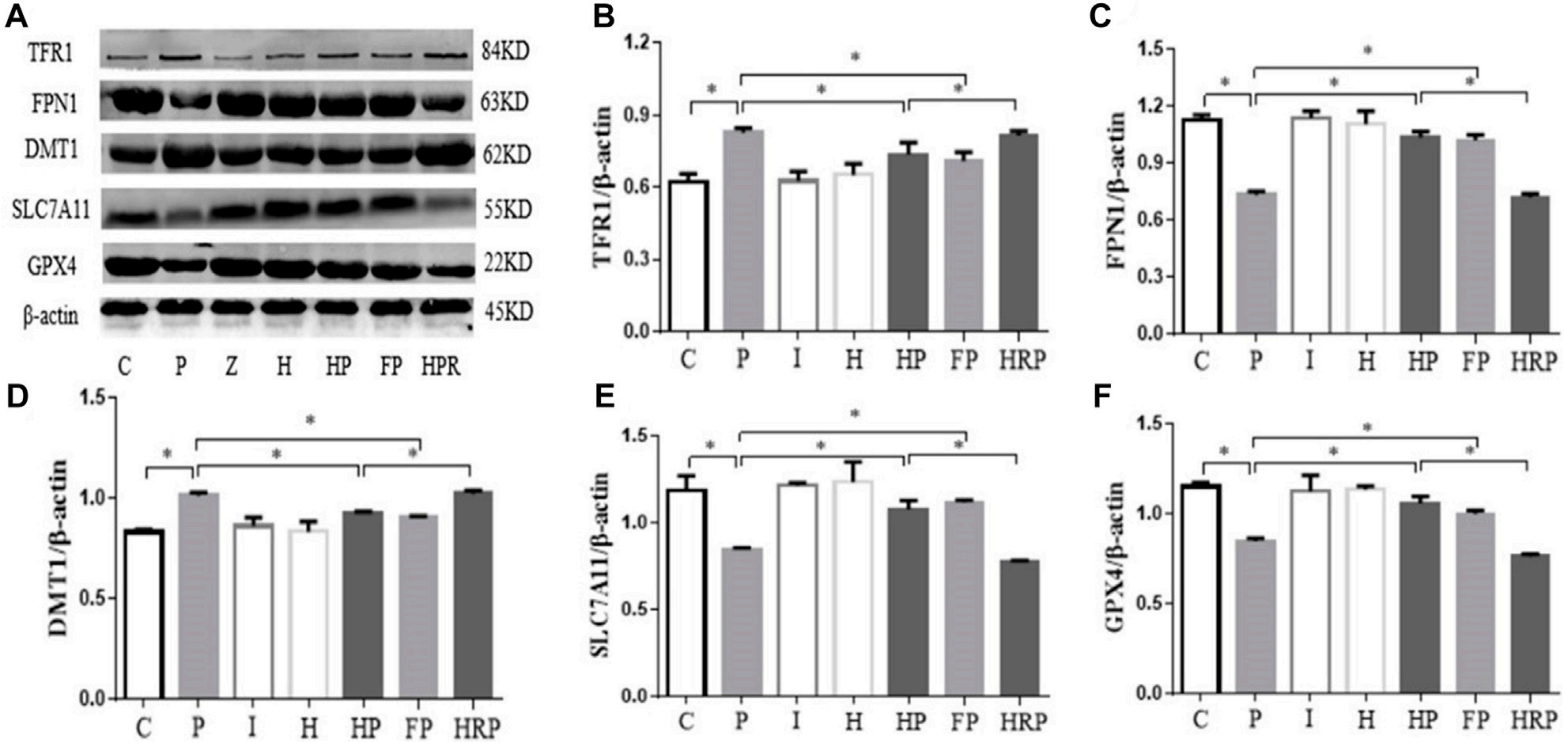
Protein expression levels of TFR1, FPN1, DMT1, SLC7A11 and GPX4 in primary hippocampal neurons exposed to propofol with or without hypoxic preconditioning, inhibitor or agonist. **(A)** TFR1, FPN1, DMT1, SLC7A11 and GPX4 protein expression was detected by Western blot analysis. **(B–F)** Quantitative analysis of the ratio of TFR1, FPN1, DMT1, SLC7A11 and GPX4 to β-actin (one-way ANOVA, *p < 0.05).

We also detected the expression of ferroptosis-related proteins by Western blot analysis. Compared to Group C, the protein expression of GPX4, SLC7A11 and FPN1 was significantly decreased in Group P, but the protein expression of DMTI and TFR1 was significantly increased in Group P. In addition, there were no significant differences in GPX4, SLC7A11, FPN1, DMT1 and TFR1 protein expression in Group I and Group H (Figure 9). Compared to Group P, the protein expression of GPX4, SLC7A11 and FPN1 was significantly increased in Groups HP and FP, and the protein expression of DMTI and TFR1 was significantly decreased in Groups HP and FP (Figure 9). Compared to Group HP, the protein expression of GPX4, SLC7A11 and FPN1 was significantly decreased in Group HRP, and the protein expression of DMTI and TFR1 was significantly increased in Group HRP (Figure 9).

## Discussion

General anesthesia provides sedation, analgesia and muscle relaxation effects. General anesthesia inhibits the autonomic nervous reactions caused by surgical operation, allowing infants and children to safely undergo surgery or nonsurgical operations, such as medical imaging examination and interventional therapy. In the last 30 years, however, many laboratory and basic animal studies have shown that general anesthetics may have toxic effects on the immature brain, destroy the morphological structure of neurons in the newborn animal brain, promote neuronal apoptosis or death, adversely affect neural development and impair long-term learning and memory function (Ing et al., 2017; Lu et al., 2017).

Among anesthesiologists and scientists worldwide, anesthetic-induced developmental neurotoxicity has raised concerns about the safety of neonatal and pediatric anesthesia. In 2017, the FDA emphasized that long-term or repeated use of general anesthetics may have some effects on children’s neurodevelopment (Andropoulos Greene, 2017). At present, a number of high-quality, multicenter, large-sample clinical studies are analyzing the effects of general anesthetics on the long-term development, learning and cognitive function of infants and young children (Sun et al., 2012; Gleich et al., 2015; Davidson et al., 2016). Because many newborns and infants with congenital defects and acquired diseases require surgical treatment, the use of general anesthetics is inevitable. Therefore, how to protect the development of the newborn brain and reduce the damage caused by anesthetics has become a hot topic in anesthesiology, neuroscience and drug toxicology research.

Propofol, as one of the most commonly used intravenous anesthetics in the clinic, has a sedative and inhibitory effect on excessive stress. The anesthetic effect of propofol is mainly related to activation of the γ-aminobutyric acid (GABA) type A receptor and inhibition of the N-methyl-D-aspartate receptor (NMDA) (Shishido et al., 1997; Forman and Miller, 2016). The balance between GABA receptor activation and NMDA receptor inhibition maintains the survival and apoptosis of neurons (Romuk et al., 2016). Previous studies have shown that propofol reduces the oxygen metabolism rate of the brain, which has a brain-protective effect (Engelhard et al., 2004; Wei et al., 2012; Wang et al., 2016; Zhang et al., 2016). However, in recent years, an increasing number of studies have shown that propofol is neurotoxic (Shibuta et al., 2022; Zhang et al., 2022), induces central neurotoxicity in developing animals and may lead to long-term learning and cognitive dysfunction (Chen et al., 2016; Li et al., 2019; Zhou et al., 2021). Our previous animal and cell experiments have shown that propofol has neurotoxicity, which not only leads to synaptic plasticity changes, decreased viability of hippocampal nerve cells, decreased survival rate of hippocampal nerve cells and increased apoptosis but also leads to abnormal development of neural networks. These effects may persist into adulthood, further causing long-term learning and cognitive dysfunction (Wan et al., 2021; Miao et al., 2022).At present, there is not enough evidence to extrapolate the findings of animal studies to humans, and more data are needed to draw a definitive conclusions about the risk of propofol exposure in infants and young children.

The rapid development period of the human brain begins at the beginning of pregnancy and continues until the age of three (Larsen and Luna, 2018), when it is vulnerable to the effects of general anesthetic drugs. Clinical retrospective studies have shown that exposure to anesthetics during early brain development is an important risk factor for behavioral and developmental disorders in children (DiMaggio et al., 2009; Kalkman et al., 2009). Therefore, primary hippocampal neurons of developing rats were used in the present study.

In the present study, treatment of hippocampal neurons with 100 μM propofol decreased the hippocampal neuron viability, mitochondrial membrane potential and ATP content as well as significantly increased hippocampal neuron ROS content and apoptosis rate (Figure 2 and Figure 3 Figure 4), which indicated that propofol had neurotoxic effects. The neurotoxicity of propofol may occur through activation of oxidative stress, damage to the mitochondria of hippocampal neurons and inhibition of ATP production, leading to a decrease in hippocampal neuron mitochondrial membrane potential and eventually to apoptosis. When hippocampal neurons were pretreated with hypoxia before incubation with 100 μM propofol, the hippocampal neuron viability, mitochondrial membrane potential and ATP content were significantly increased, and the hippocampal neuron ROS content and apoptosis rate were significantly decreased (Figures 2–4). These results suggested that hypoxia preconditioning may play a neuroprotective role by inhibiting oxidative stress caused by propofol, increasing mitochondrial membrane potential, promoting ATP generation and alleviating mitochondrial damage.

Iron is one of the most abundant and indispensable trace elements in the body. Iron is an important part of many metabolic pathways and has a variety of important physiological functions. Ferroptosis is an iron-dependent form of programmed cell death discovered by Dolma (Dolma et al., 2003) and named by Dixon (Dixon et al., 2012), and it is distinguished from apoptosis, necrosis, autophagy and pyroptosis. The essence of ferroptosis is the imbalance between the production and degradation of intracellular lipid reactive oxygen species. When the antioxidant capacity of cells is reduced, the accumulation of lipid reactive oxygen species leads to an imbalance in intracellular redox reactions, ultimately inducing cell death (Xu et al., 2020).

Extracellular Fe^3+^ binds to transferrin (TF), which interacts with transferrin receptor 1 (TFR1) on the cell membrane and is internalized *via* endosomes to the nucleus where Fe^3+^ is further reduced to Fe^2+^. Finally, Fe^2+^ is released from nuclear endosomes into the intracytoplasmic unstable pool of iron by divalent metal transporter 1 (DMT1). Excess Fe^2+^ is released outside of the cell through ferroportin 1 (FPN1) on the cell membrane to maintain iron homeostasis. The abnormal expression or dysfunction of these iron-related proteins causes an imbalance of intracellular iron ions and plays a key role in cell proliferation and differentiation.

The cystine/glutamate antiporter (System XC-) is a Na^+^-dependent amino acid antitransporter widely distributed in the phospholipid bilayer of biological cells. System XC- is composed of light chain solute carrier family seven member 11 (SLC7A11) and heavy chain solute carrier family three member 2 (SLC3A2), and it ingests a molecule of cystine and discharges a molecule of glutamate (Pitman et al., 2019). Intracellularly, cystine is first reduced to cysteine and then synthesized to glutathione (GSH) under γ-glutamyl cysteine synthetase and glutathione synthetase. GSH plays a crucial role in antioxidative stress, reducing lipid peroxidation and protecting tissues and cells. Glutathione peroxidase (GPX4) is the only enzyme in the body that effectively reduces lipid peroxides in biofilms. When System XC- is blocked, glutamate and cystine are not interchangeable, leading to intracellular glutamate accumulation, decreased GSH synthesis, decreased GPX4 activity and lipid oxides that cannot be timely reduced and then oxidized by Fe^2+^, generating a large number of lipid radicals and ROS. Because the cell membrane and plasma membrane are rich in polyunsaturated fatty acids, lipid radicals cause a cascade of reactions, which further leads to the thinning of the cell membrane and plasma membrane as well as the loss of barrier effects. Intracellular ROS immediately exacerbate destruction of the cell membrane and plasma membrane, thereby forming holes in the membrane. Moreover, cell homeostasis disorders (Galluzzi et al., 2012; Magtanong et al., 2016) activate more serious biochemical reactions. Lipid radicals damage the lipid structure of cells, and the produced lipid peroxidation products [such as 4-hydroxy-nonenal and malondialdehyde (MDA)] continue to react, constantly destroying the cell, which leads to irreversible damage to the structure and function of the cell membrane and plasma membrane, thereby causing ferroptosis of cells (Sato et al., 2018). Silencing SLC7A11 renders HT-1080 cells more sensitive to erastin-induced ferroptosis, while overexpression of SLC7A11 in HT-1080 cells significantly enhances the tolerance to ferroptosis (Chang et al., 2017). System XC- plays an important role in ferroptosis of cells.

The present study demonstrated that treatment of hippocampal neurons with 100 μM propofol significantly decreased their viability, mitochondrial membrane potential and ATP content but significantly increased the ROS content and apoptosis rates (Figure 2 and Figure 3 Figure 4). Moreover, western blot analysis showed that the protein expression levels of TFR1 and DMT1 were significantly increased but that the protein expression levels of GPX4, SLC7A11 and FPN1 were significantly decreased after treatment with 100 μM propofol (Figure 5). When hippocampal neurons were incubated with 100 μM propofol after hypoxic preconditioning, the viability, mitochondrial membrane potential and ATP content of hippocampal neurons were significantly increased, and the ROS content and apoptosis rate were significantly decreased (Figures 2–4). Western blot analysis showed that the protein expression levels of TFR1 and DMT1 were significantly decreased, while the expression levels of GPX4, SLC7A11 and FPN1 were significantly increased after incubation with 100 μM propofol after hypoxic preconditioning (Figure 5).

**FIGURE 10:**
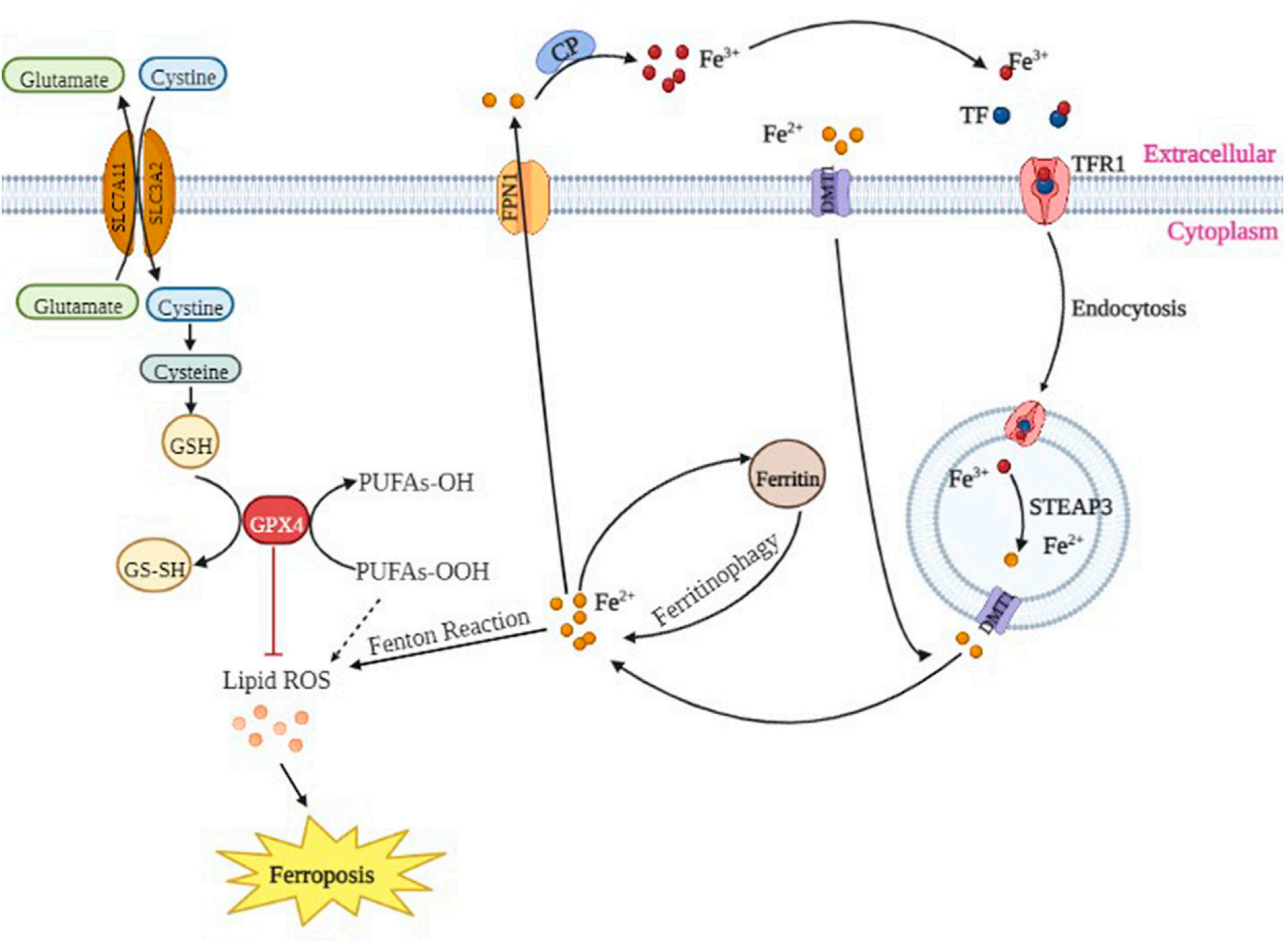
The molecular mechanisms of ferroptosis.

Therefore, these findings suggested that the neurotoxicity of propofol may be related to ferroptosis. Propofol may reduce the activity or expression of GPX4 protein by decreasing the expression of SLC7A11 protein, ultimately leading to failure of timely reduction of intracellular lipid oxides. At the same time, propofol may increase the expression of TFR1 and DMT1 proteins; a large amount of Fe^3+^ enters the cells, while FPN1 protein is decreased. Because excess Fe^2+^ cannot be transported out of the cells in time, it reacts with intracellular lipid oxides, produces a large amount of lipid radicals and ROS, ultimately activating ferroptosis. Hypoxic preconditioning may increase the activity of GPX4 protein by increasing the expression of SLC7A11 protein or by increasing the expression of GPX4 protein, thereby allowing the intracellular lipid oxide to be reduced in time. At the same time, hypoxic preconditioning may decrease the protein expression of TFR1 and DMT1 as well as control the entry of Fe^3+^ into cells and increase the expression of FPN1 protein, which allows excess Fe^2+^ to be transported out of cells in time to inhibit ferroptosis, thereby alleviating the neurotoxicity of propofol and playing a neuroprotective role (Figure 10).

To test this hypothesis, we treated primary hippocampal neurons with propofol, ferroptosis agonists, ferroptosis inhibitors and hypoxic preconditioning. Treatment of hippocampal neurons with 100 μM propofol significantly decreased the viability, mitochondrial membrane potential and protein expression levels of GPX4, SLC7A11 and FPN1 but also significantly increased the protein expression levels of DMT1 and TFR1 (Figure 6; Figures 8A, B; Figure 9). When hippocampal neurons were pretreated with hypoxic preconditioning or the ferroptosis inhibitor (ferrostatin-1, 2 μM) followed by incubation with 100 μM propofol, the viability, mitochondrial membrane potential and protein expression levels of GPX4, SLC7A11 and FPN1 were significantly increased, but the protein expression of DMT1 and TFR1 was significantly decreased (Figure 6; Figures 8A, B; Figure 9). When hippocampal neurons were pretreated with a ferroptosis agonist (RSL3, 10 μM) and then hypoxic preconditioning followed by incubation with 100 μM propofol, the viability, mitochondrial membrane potential and protein expression of GPX4, SLC7A11 and FPN1 were significantly decreased, but the protein expression levels of DMT1 and TFR1 were significantly increased (Figure 6; Figures 8A, B; Figure 9). Thus, these findings indicated that propofol has neurotoxic effects and activates ferroptosis. The neurotoxicity of propofol may be related to the activation of ferroptosis. Hypoxic preconditioning has a neuroprotective effect, which reduces the neurotoxicity of propofol, and it also inhibits or alleviates the ferroptosis caused by propofol. Therefore, the reduction in the neurotoxicity of propofol by hypoxic preconditioning may be related to the inhibition of ferroptosis.

Studies have added Fe^2+^ to the culture medium of spinal neurons and found that the level of Fe^2+^ is positively correlated with the production of peroxidation products, such as ROS and malondialdehyde (MDA), while the activity of neurons is negatively correlated with the content of ROS and MDA, indicating that Fe^2+^ and ROS are promoters and mediators of ferroptosis (Smith et al., 2016). Previous studies have confirmed that ferrous ions catalyze the formation of a large number of ROS, namely, Fe^2+^ + H_2_O_2_ → Fe^3+^ + (OH)^-^ + **·**OH**,** Fe^3+^ + O_2_^−^→ Fe^2+^ + O_2_, which is the famous Fenton reaction (Ayala et al., 2014) (Figure 10). Other studies have found that antioxidants (such as α-vitamin E and β-carotene) and an iron chelating agent (deferoxamine) significantly inhibit erastin-induced cell ferroptosis (Dixon et al., 2012). In addition, MDA content is positively correlated with ferroptosis, indicating that iron homeostasis and lipid peroxidation are key links in the occurrence of cell ferroptosis. Superoxide dismutase (SOD) is a metalloenzyme that widely exists in animals, plants and microorganisms. SOD catalyzes the disproportionation reaction of superoxide radicals (O^2-^) in organisms, and it is a natural eliminating agent of O^2-^ in organisms. Thus, scavenging O^2-^ plays an important role in the self-protection system of organisms. Acyl coenzyme A synthetase long chain member 4 (ACSL4), a member of the ACSL family, catalyzes the synthesis of acyl CoA *in vivo* (Doll et al., 2017; Cheng et al., 2020). ACSL4 has been found to be a key gene in the ferroptosis (Yuan et al., 2016). ACSL4 can promote the activation of long-chain unsaturated fatty acids, and when ACSL4 is missing, it can lead to the oxidation of long-chain unsaturated fatty acids, inducing ferroptosis (Ma et al., 2020).

The present study found that when primary hippocampal neurons were incubated with 100 μM propofol at 37°C for 3 h, the content of SOD was significantly decreased, and the contents of ROS, Fe^2+^ and MDA were significantly increased (Figure 7; Figures 8D–F). When hippocampal neurons were pretreated with hypoxic preconditioning or a ferroptosis inhibitor (ferrostatin-1, 2 μM) and then incubated with 100 μM propofol, the SOD content was significantly increased, while the.

ROS, Fe^2+^ and MDA contents were significantly decreased (Figure 7; Figures 8D–F). When hippocampal neurons were pretreated with a ferroptosis agonist (RSL3, 10 μM) and then hypoxic preconditioning followed by incubation with 100 μM propofol, we found that the SOD content was significantly decreased, while the contents of ROS, Fe^2+^ and MDA were significantly increased (Figure 7; Figures 8D–F). Thus, these findings demonstrated that propofol activates ferroptosis and that hypoxic preconditioning alleviates the propofol-induced ferroptosis.

In summary, the present findings suggested that the neurotoxicity of propofol in developing rats may be related to ferroptosis. Propofol may induce neurotoxicity by activating ferroptosis and eventually leading to neuronal apoptosis, while hypoxic preconditioning may reduce the neurotoxicity of propofol by inhibiting ferroptosis and reduce neuronal apoptosis to produce neuroprotective effects.

Although hypoxic preconditioning is a promising treatment option in the field of neuroprotection, there are still many problems to be solved. For example, the optimal solution for neuroprotection and repairment induced by hypoxic preconditioning and how much the degree of hypoxia is adaptive has not been determined, so our research team will continue to work hard.

## Data Availability

The original contributions presented in the study are included in the article/supplementary material further inquiries can be directed to the corresponding authors.
